# Non-hispanic whites have higher risk for pulmonary impairment from pulmonary tuberculosis

**DOI:** 10.1186/1471-2458-12-119

**Published:** 2012-02-10

**Authors:** Jotam G Pasipanodya, Edgar Vecino, Thaddeus L Miller, Guadalupe Munguia, Gerry Drewyer, Michel Fernandez, Philip Slocum, Stephen E Weis

**Affiliations:** 1 Department of Internal Medicine, UNT- Health Science Center at Fort Worth, Fort Worth, TX, USA; 2 Department of Internal Medicine, Division of Infectious diseases, UT Southwestern Medical Center at Dallas, Dallas, Texas, USA; 3 Department of Internal Medicine, A.T. Still University of Health Sciences, Kirksville, MO, USA; 4 Tarrant County Public Health Department, Division of TB Elimination, 1101 S. Main Street, Fort Worth, TX, USA

## Abstract

**Background:**

Disparities in outcomes associated with race and ethnicity are well documented for many diseases and patient populations. Tuberculosis (TB) disproportionately affects economically disadvantaged, racial and ethnic minority populations. Pulmonary impairment after tuberculosis (PIAT) contributes heavily to the societal burden of TB. Individual impacts associated with PIAT may vary by race/ethnicity or socioeconomic status.

**Methods:**

We analyzed the pulmonary function of 320 prospectively identified patients with pulmonary tuberculosis who had completed at least 20 weeks standard anti-TB regimes by directly observed therapy. We compared frequency and severity of spirometry-defined PIAT in groups stratified by demographics, pulmonary risk factors, and race/ethnicity, and examined clinical correlates to pulmonary function deficits.

**Results:**

Pulmonary impairment after tuberculosis was identified in 71% of non-Hispanic Whites, 58% of non-Hispanic Blacks, 49% of Asians and 32% of Hispanics (*p *< 0.001). Predictors for PIAT varied between race/ethnicity. PIAT was evenly distributed across all levels of socioeconomic status suggesting that PIAT and socioeconomic status are not related. PIAT and its severity were significantly associated with abnormal chest x-ray, *p *< 0.0001. There was no association between race/ethnicity and time to beginning TB treatment, *p *= 0.978.

**Conclusions:**

Despite controlling for cigarette smoking, socioeconomic status and time to beginning TB treatment, non-Hispanic White race/ethnicity remained an independent predictor for disproportionately frequent and severe pulmonary impairment after tuberculosis relative to other race/ethnic groups. Since race/ethnicity was self reported and that race is not a biological construct: these findings must be interpreted with caution. However, because race/ethnicity is a proxy for several other unmeasured host, pathogen or environment factors that may contribute to disparate health outcomes, these results are meant to suggest hypotheses for further research.

## Background

Health outcome disparities associated with race and ethnicity are well documented for many diseases and patient populations. While there are a variety of explanations for these effects, they are not fully understood [1-3]. Socio-economic, biological, cultural, demographic, and other factors all contribute to an individual's health before, during and after illness [1,2,4]. While some contributors to health disparities are well defined the contribution of biological and gender differences, personal behaviors, value choices, and race/ethnicity on specific diseases and their clinical outcomes are not [1,3].

It is well established that tuberculosis (TB) is disproportionately prevalent among economically disadvantaged and racial/ethnic minority populations [5-8]. The health impacts of TB associated with differences in race, ethnicity, and more primary health risks are incompletely known [5-12]. In a prior study, we measured the frequency and degree of pulmonary impairment in TB patients who were treated with standard regimes delivered by directly observed therapy (DOT) [13]. Spirometry-defined pulmonary impairment after tuberculosis (PIAT) was found in a majority of the cohort, and was more common in US born and older patients [13,14]. The study's sample size did not allow stratified analysis of PIAT prevalence and severity between race/ethnic and other patient groups. We expanded our sample to allow a comparison of PIAT frequency across self-identified race/ethnicity groups and by socioeconomic status.

## Methods

### Patients and setting

This was a prospective cohort study of all patients 16 years of age and older receiving treatment for culture-confirmed pulmonary tuberculosis at Tarrant County Public Health (TCPH) from July 2005 to December 2009. The population includes all persons with culture-confirmed pulmonary tuberculosis in Tarrant County, some of whom also had concurrent extra-pulmonary tuberculosis. Texas requires all diagnosed TB cases be reported to the local public health authorities [15]. TCPH is the health authority for an urban county with a 2010 population of 1,789,900 [15]. TCPH provides treatment for all persons with TB within this jurisdiction, using universal DOT delivered to the patient's preferred location [15,16]. All patients were treated with standard 4 drug American Thoracic Society (ATS) and Centers for Diseases and Prevention Control (CDC) recommended anti-TB regimens [17]. Patients who had completed at least 20 weeks of this treatment were asked to participate in this study of their pulmonary function. The Institutional Review Board of the University of North Texas Health Science Center at Fort Worth approved the study; IRB project #24-109. All subjects gave written informed consent.

### Pulmonary function testing

Pulmonary function tests (PFTs) by spirometry were performed on consenting patients. Spirometry was conducted according to ATS guidelines for maneuver, techniques and quality control using the Spirotouch device (Spirotouch Spirometry System 086578; Spacelabs Burdick; Deerfield, WI) [18,19]. Patients with a history of bronchodilator use received nebulized albuterol 15 min before the test. Consistent results were considered variation of 5% or less between measurements on three separate tests. The best of three consistent results was used to grade pulmonary function.

Impairment was defined and graded using American Medical Association (AMA) guides for evaluation of permanent impairments [20]. Forced Expiratory Volume in 1 min (FEV1) > = 80%, Forced Vital Capacity (FVC) > = 80% and FEV1/FVC > 70% of predicted were considered normal. Other results defined pulmonary impairment. Impairment was categorized as none, mild (if FEV1 or FVC was > 60% but < 80%), moderate (if FEV1 or FVC was 41% to 59%) or severe (if FEV1 or FVC was < 40%) using an interpretive algorithm from the AMA [18-20].

Trained research personnel obtained demographic data from patients at the time of enrolment using a standardized instrument. Data were double entered into a Microsoft Office 2003 ACCESS database (Microsoft Corporation, Redmond, WA. 98052). Subjects self-identified their race/ethnicity, and were given an option to identify themselves as Hispanic in accordance with US federal definitions [21]. Because of their small numbers we combined self-identified Pacific Islanders, Native American Indians, and Arabs into one group.

Socioeconomic status was assessed according to established methods [22,23] and included (1) highest level of education attained, (2) employment status at diagnosis, (3) self-identified occupation, and (4) estimate of household income. Education was categorized into quartiles of years < 12, 12, 12 to 15 and > 16 years. Similarly, area-median household income, derived from census-tract ZIP codes of the patient's home address, was divided into quartiles of < $27,250, $27,251 to $37 180, $37,180 to 52,777 and > $52,778; ranges comparable to published data from US TB patients [5,24]. Homeless person who did not report income were treated as missing data. We scored patients' occupations using standard methods and correlated them to levels of education [22,23,25]. Occupational status was ranked according to prestige [22,23,25]. Education was then used as a proxy for socioeconomic status [23].

Time to beginning TB treatment, defined as the time from self-reported onset of symptoms to beginning tuberculosis therapy, was measured to give insight into patient-related factors associated with accessing healthcare [1]. Ever smokers were patients who gave a history of current or past cigarettes smoking. Lifetime volume of cigarette exposure was estimated using pack-years. Exposure to solid fuel smoke (biomass exposure) and duration of biomass exposure was compared between groups.

We correlated radiographic abnormality with pulmonary function using a validated scoring rubric derived from published sources (Table 1) [26]. An experienced physician (SEW) read the baseline chest x-rays taken during therapy and follow-up chest x-rays taken after 20 weeks of treatment. TB disease site was classified as "pulmonary only" or "both pulmonary and extra pulmonary. Observed abnormalities, cavitation, and infiltration were standardized and scored using the rubric. The summed total score was correlated with observed pulmonary function.

**Table 1:** Rubric to standardize chest radiographic findings

Findings	Score
**Abnormal Appearance**	

No	0

Yes	1

**Cavitation**	

None	0

Cumulative diameter less than 2 cm	1

Cumulative diameter 2 to 4 cm	2

Cumulative diameter greater than 4 cm	3

**Extent and pattern of infiltrating lesions**	

None	0

Occupy less than 25% of thoracic cavity	1

Occupy 25 to 49% of thoracic cavity	2

Occupy more than 50% of thoracic cavity	3

Miliary pattern	1

### Statistical analysis

Parsimonious multivariate logistic regression models were constructed and analyzed for the full sample and separately for US-born, foreign-born persons and each racial/ethnic group. Both age and smoking have been shown to independently exacerbate pulmonary function decline so were included in all multivariate models [27-29]. The median age at which impairment and moderate/severe impairment occurred among the racial/ethnic groups were compared using Kaplan-Meir methods. Comparison between groups was performed using Chi-Square or Fisher's exact tests and/or analysis of variance (ANOVA) plus the Kruskal-Wallis tests when appropriate. Analysis was performed using SPSS version 12 for Windows (SPSS Inc; Chicago, IL) and GraphPad Prism version 5 (GraphPad Software; La Jolla, CA).

## Results

**Figure 1:**
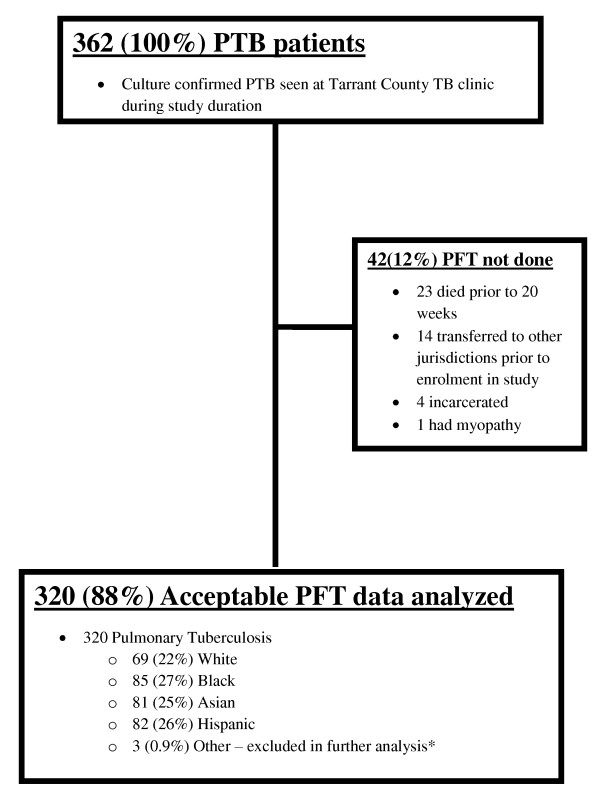
**Study enrolment**.

Between July 2005 and December 2009, 362 patients with culture confirmed pulmonary tuberculosis were reported to Tarrant County Health and were eligible for study enrolment (Figure 1). Of these, 320 (88%) were enrolled. Sixty-nine (22%) self-identified as non-Hispanic White, 85 (27%) as non-Hispanic Black, 81 (25%) as Asian, 82 (26%) as Hispanic and 3 (0.9%) were combined as "other" racial/ethnic group. The 3 subjects in the "other" racial/ethnic group were all male and included two with mild impairment and one non-impaired and were excluded from further analysis.

TB disease type and site, and patients' access to TB care was similar between race/ethnicity (Table 2). There were significantly different demographic and clinical characteristics between race/ethnicity (Table 2). HIV infection was significantly higher among non-Hispanic Blacks and level of education significantly lower among Hispanics compared to non-Hispanic Whites. Clinical and demographic characteristics, including age and smoking of US-born were significantly different from those who were foreign-born. Both proportion of ever-smokers and level of lifetime cigarette use was significantly higher among Whites (*p *< 0.001 for both measures) than other groups (Table 2).

**Table 2:** Demographic and clinical characteristics of 317 patients with pulmonary tuberculosis (TB) included in the analysis

	Non-Hispanic White	Non-Hispanic Black	Asian	Hispanic	
	
	n = 69	n = 85	n = 82	n = 81	p-value
**Demography**					
Male	58 (84)	54 (64)	58 (71)	52 (64)	0.023
US-born	66 (96)	62 (73)	4 (5)	11 (4)	< 0.001
Foreign-born	3 (4)	23 (27)	78 (95)	70 (86)	
Age (mean[SD]) years	54.33 (13.10)	43.71 (13.51)	44.91 (16.61)	45.95 (15.81)	< 0.001
**Clinical**					
HIV positive	5 (7)	15 (18)	3 (4)	7 (9)	0.052
Ever-Smokers n(%)	56 (81)	44 (52)	35 (43)	39 (48)	< 0.001
Smoking volume(mean[SD]) pack-years	32.68 (39.56)	8.19 (15.48)	5.52 (9.50)	4.67 (11.78)	< 0.001
Biomass Smoke					
Exposed n (%)	5 (9)	10 (15)	29 (45)	28 (38)	*0.001*
Biomass Smoke Exposure duration					
(mean [SD]) years	0.80 (3.26)	2.52 (7.38)	6.94 (12.54)	7.09 (14.27)	*0.001*
FVC (% predicted [SD])	77.54 (23.70)	78.69 (19.05)	82.09 (19.10)	90.15 (21.19)	0.001
FEV1 (% predicted[SD])	71.12 (24.30)	76.98 (22.69)	82.85 (19.54)	91.83 (23.03)	< 0.001
FEV1/FVC (% [SD])	73.13 (13.48)	81.11 (13.35)	84.01 (9.75)	85.26 (10.01)	< 0.001
BMI (mean[SD])	21.23 (5.24)	23.07 (4.58)	22.32 (4.90)	25.08 (8.62)	< 0.001
**Disease site and pattern**					
PTB only	66 (96)	75 (88)	76 (93)	75 (93)	0.39
PTB and EPTB	3 (4)	10 (12)	6 (7)	6 (7)	
Pattern of Impairment					
Restrictive	33 (68)	34 (69)	30 (75)	21 (81)	0.571
Obstructive	8 (16)	4 (8)	3 (8)	3 (11)	
Mixed	8 (16)	11 (23)	7 (17)	2 (8)	
Access (median [IQR]) Days to Begin TB Treatment	63 (183)	65 (130)	93 (157)	80 (103)	0.978

n(%) denotes counts and column percentage, unless indicated otherwise; mean[SD] = mean and standard deviation; median[IQR] = median and inter-quartile range. *BMI *body mass index (kg/m^2^); *EPTB *extra-pulmonary TB; *HIV *human immunodeficiency virus; *FEV1 *force expiratory volume in 1 second; *FVC *forced vital capacity. Three patients designated 'other' racial group who were enrolled in study were not included in analysis.

**Figure 2:**
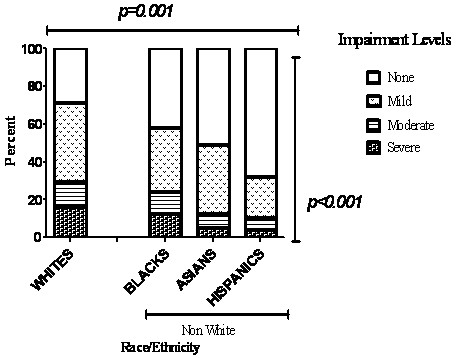
**Comparisons of frequency and severity of pulmonary impairment between 317 self-identified racial and ethnic groups comprising 69 non-Hispanic Whites, 85 non-Hispanic Blacks, 82 Asians and 81 Hispanics**. Figure 2 demonstrates that proportions impaired and the severity of impairment significantly varies between racial/ethnic groups; specifically both impairment frequency and severity was significantly higher among Whites compared to non-Whites.

**Figure 3:**
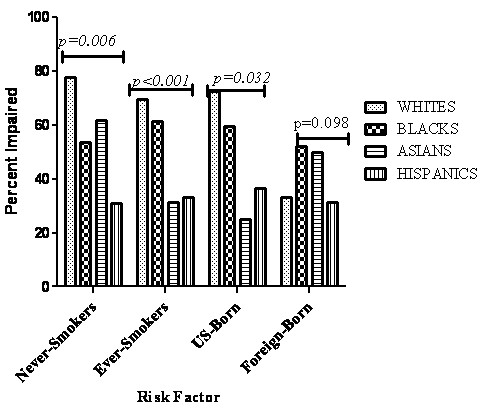
**Comparison of the frequency of pulmonary impairment among all self-identified racial groups by country of birth and smoking status**.

**Figure 4:**
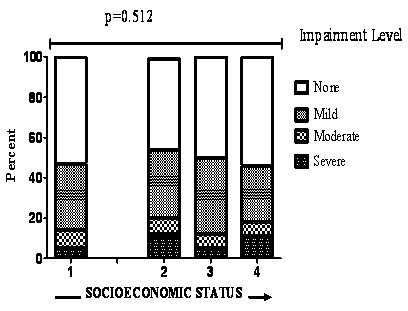
**Comparisons of the frequency and severity of pulmonary impairment among patient with different socioeconomic status**. Figure 4 shows that proportions impaired and the severity of impairment does not vary with increase in socioeconomic status.

The distribution of pulmonary impairment after tuberculosis (PIAT) and its severity among racial/ethnic groups, by smoking status and by socioeconomic status is shown in Figures 2, 3, and 4, respectively. PIAT was more frequent among non-Hispanic Whites compared to other race/ethnic groups *(p < 0.001)*, and was more severe (*p *= 0.001) (Figure 2). Pulmonary impairment was identified in 71% of non-Hispanic Whites, 58% of non-Hispanic Blacks, 49% of Asians and 32% of Hispanics. PIAT frequency was significantly higher among non-Hispanic Whites compared to other racial/ethnic groups in both ever-smokers and never-smokers, (*p *< 0.0001) (Figure 3).

The distribution of employment, income, occupation, and education data among subjects was similar to that reported for other US TB patients (9-11). Education and income were significantly correlated (Pearson's correlation coefficient (r) = 0.21, *p *< 0.001). When occupational status was ranked according to prestige, it also significantly correlated with both education and income (r = 0.33, *p *< 0.001 and r = 0.15, *p *= 0.005, respectively). PIAT prevalence was evenly distributed across all levels of socioeconomic status: when the highest level of education attained was used as a proxy for socioeconomic status (Figure 4).

The median "time to beginning TB treatment" for non-impaired persons was 62 days (interquartile range [IQR] was 12-110); 93 days for mildly impaired persons (IQR 61-110), 138 days for moderately impaired subjects (IQR 32-271), and 37 days for severely impaired subjects (IQR 12-60). There was no significant association between race/ethnicity and time to beginning TB treatment, (p = 0.978) (Table 2). Similarly, no association between time to beginning treatment and PIAT was observed (p = 0.058) (data not shown).

**Figure 5:**
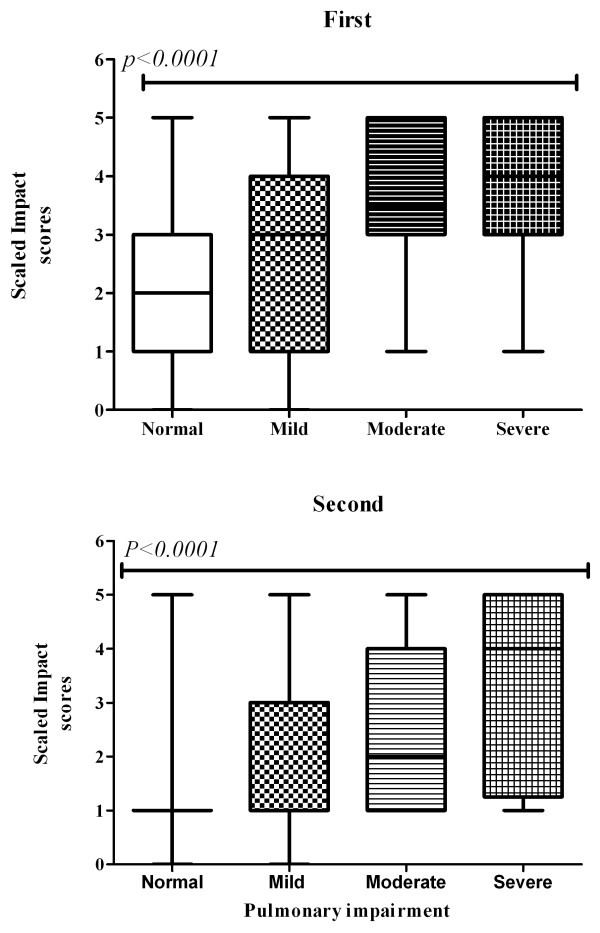
**Distribution and severity of lung damage and baseline chest x-ray (first)**. Distribution and severity of lung damage at subsequent chest x-ray (second).

We obtained baseline chest x-ray results for 99% of subjects (n = 314), and for 90% (n = 254) of subjects after either 20 weeks or at therapy completion. Pulmonary impairment was significantly (*p *< 0.001) correlated with the presence and magnitude of abnormal chest x-ray findings for both baseline (Spearman's correlation coefficient (r) = 0.4), and subsequent readings, (r = 0.42). Figure 5 shows the distribution of a standardized severity index among subjects with pulmonary impairment identified by spirometry.

In univariate analysis race/ethnicity, age and US-birth were significantly associated with PIAT (Table 3). The likelihood of PIAT increased by 2% (95% confidence interval [CI] 1, 3) for each 1 year increase in age. PIAT was 2.3 times more common (95% CI 1.46, 3.61) in US-born than foreign-born subjects. Race/ethnic groups and foreign birth were correlated: Spearman's r = 0.69, *p < 0.001*.

**Table 3:** Unadjusted odds ratio for some pulmonary impairment

	OR (95% C.I)	*p*-value
**Race**		***< 0.001***
Whites	5.18 (2.58, 10.42)	
Blacks	2.88 (1.53, 5.43)	
Asian	2.02 (1.07, 3.81)	
Hispanics (reference)	**	
**Access**		
Days to Begin TB treatment		
(days)	1.00 (1.00, 1.01)	0.098
**Demographic and clinical characteristics**		
Male	1.36 (0.84, 2.21)	0.207
Females (reference)*		
US-born	2.30 (1.46, 3.61)	***< 0.001***
Foreign-born(reference)*		
Ever-Smokers	1.00 (0.64, 1.56)	0.997
Never-Smokers (reference)*		
Biomass Smoke Exposure	1.33 (0.77, 2.28)	0.308
No Biomass Smoke Exposure (reference)*		
Smoking Volume (pack-year)	1.01 (1.00, 1.03)	***0.007***
Age (years)	1.02 (1.01, 1.03)	***0.031***
BMI (kg/m2)	0.97 (0.93, 1.01)	0.087
Socioeconomic Status		
**Education**		
Some Education (< 12 years)(reference)*	**	0.470
High School Graduate (12 years)	1.46 (0.86, 2.49)	
Some College (13 - 15 years)	1.33 (0.67, 2.64)	
College Graduate (16 or more year)	1.33 (0.64, 2.78)	
**Occupation**		
1 (most prestigious)	**	0.411
2	1.17 (0.52, 2.67)	
3	1.68 (0.90, 3.14)	
4 (least prestigious)	1.41 (0.78, 2.56)	
**Area-median household income**		
< US$27 270 (reference)	**	0.408
US$27 271 - 37 180	0.82 (0.46, 1.46)	
US$37 181 - 52 777	0.76 (0.40, 1.44)	
US$52 777	1.55 (0.82, 2.92)	

*BMI *body mass index (kg/m^2^); *OR *odds ratio; *CI *confidence interval

In a multivariate analysis that controlled for potential demographic and clinical confounders; the only significant predictor for PIAT was non-Hispanic White race/ethnicity, among whom PIAT prevalence was 3 times greater (95% C.I. 1.18, 8.40). Since race/ethnic group and foreign birth were significantly correlated, and to avoid confounding, separate multivariate regression models were constructed and are shown in Tables 4 and 5. Risk factors for impairment were variable between race/ethnicity, with age independently predicting impairment in non-Hispanic Whites and non-Hispanic Blacks (Table 4). Smoking was associated with three fold (95% CI 1.15, 7.85) increased risk for impairment among Asians, but was not predictive for impairment among non-Hispanic Whites (Table 4, Figure 3). Table 5 shows the multivariate regression model containing age, smoking and race/ethnicity of 144 US-born persons. In the model, only non-Hispanic White race/ethnicity and age independently predict PIAT. The age-related risk for PIAT increased 5% (95CI 2.0, 8.1) per year of age.

**Table 4:** Predictors for pulmonary impairment in all 69 Whites, 85 Blacks, 82 Asians and 81 Hispanics with pulmonary tuberculosis

	Non-Hispanic Whites	Non-Hispanic Blacks	Asians	Hispanics
Age (years)	1.06 (1.01, 1.11)*	1.04 (1.00, 1.08)*	0.98 (0.95, 1.01)	1.02 (0.99, 1.05)
US-born †	27.89 (1.02, 766.08)*	0.98 (0.31, 3.06)	0.28 (0.03, 3.08)	1.07 (0.27, 4.17)
Ever Smokers ‡	2.68 (0.48, 14.98)	1.09 (0.38, 3.11)	3.0 (1.15, 7.85)*	1.02 (0.39, 2.64)
Cox & Snell R-Square	0.12	0.06	0.12	0.02
Chi-Square (p-value)	0.029	0.156	0.018	0.624

*p < 0.05; † Reference = Foreign-born; ‡ Reference = Never Smokers; *OR *odds ratio; *CI *confidence interval

**Table 5:** Predictors for pulmonary impairment in 144 US- born patients with pulmonary tuberculosis

	OR (95%CI)	P-value
Age (years)	1.05 (1.02, 1.08)	0.001
Ever-Smokers*	1.77 (0.73, 4.29)	0.208
Non-Hispanic Whites †	4.94 (1.13, 21.63)	0.034
Non-Hispanic Blacks †	3.51 (0.81, 15.12)	0.093
Asians †	0.95(0.05, 18.57)	0.971
Constant	0.04	0.003

Comparison groups; *Ever-Smokers versus Never-Smokers; † Hispanics; *OR *odds ratio; *CI *confidence interval; The patients whose race/ethnicity was designated 'other' are excluded in this analysis

**Figure 6:**
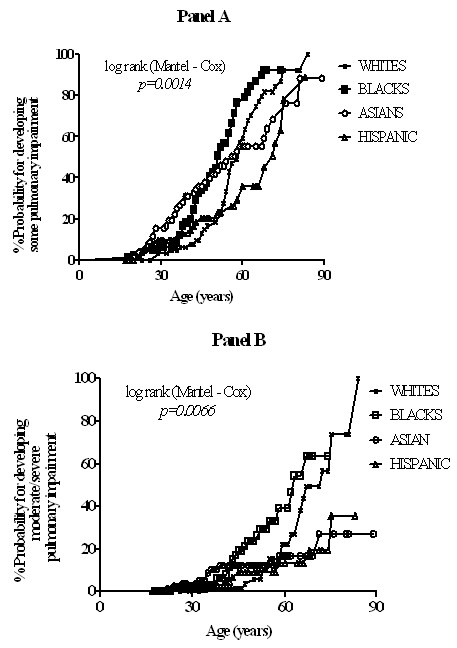
**Hazard ratios for different racial groups in developing some pulmonary impairment (Panel A.) and moderate or severe pulmonary impairment (Panel B) with increase in age**. The median ages for panel A are; non-Hispanic Whites 58 years, non-Hispanic Blacks 51 years, Asians 57 years and Hispanics 68 years. For panel B the median age for non-Hispanic Whites is 72 and that for non-Hispanic Blacks is 63.

Onset of age-related lung function decline is variable [19,30,31]; however, for this study cohort onset of impairment was related to the age at which the different race/ethnic groups acquired tuberculosis. Consequently, the risk for moderate or severe pulmonary impairment is significantly higher among older Whites compared with non-Whites. As an example, the median age was 51 years for non-Hispanic Blacks, 59 for Whites, 56 for Asians and 71 years for Hispanics (Figure 6). Similarly, the probability for developing moderate to severe impairment was higher in non-Hispanic Blacks of younger age groups compared to other race/ethnic groups (Figure 6, panel B). The median age for non-Hispanic Blacks was 63 and that for non-Hispanic Whites was 72, *p *= 0.0239. The hazard ratio [HR] was 0.45 (0.22, 0.90).

## Discussion

In the U.S., racial/ethnic minorities and foreign-born persons face disparate risks for TB infection and higher levels of poor TB disease outcomes, including mortality [5-9]. We analyzed the relationship between race/ethnicity and PIAT in a cohort with culture-confirmed pulmonary tuberculosis that had completed a minimum of 20 weeks of therapy. We found that self-identified non-Hispanic White TB patients had disproportionately more frequent and severe pulmonary impairment relative to other race/ethnicities (72% vs. 48%), odds ratio (OR) of 3.15. These differences persist despite control for the effects of age, body mass index, smoking, access to medical treatment, foreign birth and socio-economic status. Among the potential explanatory variables analyzed, only age and race/ethnicity were significant predictors for impairment in US born persons. These data demonstrate a previously unrecognized disparate negative health impact to specific populations of TB patients.

Current U.S. policy does not consider older adults high-priority candidates for testing and treatment of LTBI unless they have specific risks for developing TB disease [17,32]. These recommendations are based on the potential for adverse drug events associated with LTBI treatment. Predictors for PIAT varied between race/ethnic groups and by country of birth. We found the likelihood for PIAT to increases by an average 5% for each additional increase in age for US-born patients (Table 5; Figure 6). NHANES data showed that poorer lung function is also associated with poor clinical outcomes including premature death [30,33]. This together with our findings suggests that moderate to severe PIAT may also be associated with earlier mortality. Future versions of LTBI treatment guidelines should consider reduction of tuberculosis burden from preventing PIAT as an additional treatment benefit.

Cigarette smoking, an established cause of pulmonary impairment, was significantly more prevalent among non-Hispanic Whites compared to other racial/ethnic groups. The proportion of non-Hispanic Whites impaired among never-smokers was 70% compared to 78% among ever-smokers. PIAT was more frequently encountered among non-Hispanic Whites compared to other racial/ethnic groups *(p < 0.001)*, and when encountered was more likely to be severe (p = 0.001) (Figure 2) even after controlling for age and smoking (Figure 3, Tables 4). While there were more non-Hispanic Whites who smoked our data shows this difference is not sufficient to explain the more severe impairment found in non-Hispanic Whites.

Previous studies have investigated pulmonary sequelae of TB from a number of perspectives, but these are not readily generalized to US populations [31,34-37]. Poh et al evaluated patients hospitalized for treatment with non-rifampin chemotherapy regimens and identified older age, disease severity at presentation and heavy smoking as predictors for pulmonary impairment [36]. A population-based study from Latin America demonstrated that older age and repeated TB disease were associated with pulmonary impairment [31]. Two South African studies of patients receiving inpatient treatment [34,37] similarly demonstrated that repeated TB disease significantly increased risks for pulmonary impairment. Race/ethnicity was not explored in these studies [31,34,37. Despite management with best currently available therapy for tuberculosis we identified some PIAT in over half (52%) of patients and severe PIAT, in which less than 50% of personal lung function remains, in almost 1 in 10 patients (9%). Prevalence and severity of PIAT were not associated with diagnostic or treatment delay, suggesting that it occurs early among those with TB. Therefore, strategies to mitigate PIAT must primarily rely on prevention of active TB.

Our study failed to detect association between socioeconomic status and pulmonary impairment. This was an unexpected and novel finding. Poorer health outcomes are consistently associated with low socioeconomic status [1,5,23]. Despite Hispanics' lower socioeconomic status in our study cohort, and their higher TB incidence rates relative to other racial/ethnic groups in the US [5]; they enjoyed apparent protection against pulmonary impairment compared to other racial/ethnic groups. This finding supports what has been called the "healthy Hispanic Paradox," in which Hispanics experience disproportionately greater life expectancy relative to other racial/ethnic groups [38,39]. Equity in health care access within the study area allowed by the public treatment of TB may explain health outcomes' independence from socioeconomic status.

There are several areas within our study vulnerable to ascertainment bias: such as the fact that race/ethnicity was self-reported, identification and grading of pulmonary impairment was biased towards an obstructive pattern and that the chest x-ray grading of impairment lacks consensus of standardization. Both race and ethnicity are contextual, mutually contradictory and usually assume socially defined constructs with no biologic basis such that even the definitions used by U.S. federal agencies change with every 10-year census [2,40]. Even though mixed race/ethnicity is rare among self-identified non-Hispanic Whites, the US Hispanic population has a heterogeneous ethnic ancestry comprising of American Indian, European and African origins [41]. In addition, 30% of self-identified US-born Blacks consider themselves of mixed race [41,42]. As a result, the true effects of race/ethnicity on health outcomes may be difficult to clearly distinguish and are subject to confounding. Indeed, AMA grading is biased towards impairment that is obstructive in nature; hence patients with restrictive patterns might be under-represented in these estimates [18-20]. Given these limitations, it cannot be excluded that the findings reflect different phenotypic disease entities among different groups, of which some might be influenced by smoking and some not.

## Conclusion

In conclusion, we found that pulmonary TB patients, who self-identified as non-Hispanic White, had more prevalent and more severe pulmonary impairment. The risk for pulmonary impairment remained after several factors such as smoking and socioeconomic status were controlled. Since race/ethnicity was self reported and race is not a biological construct, these findings must be interpreted with caution. However, because since race/ethnicity is a proxy for several other unmeasured host, pathogen or environment factors that may contribute to disparate health outcomes, these results are meant to suggest hypotheses for further research. Nevertheless, if these findings are confirmed among other populations in other locations, they suggest that the decision-making thresholds of risk of TB prevention strategies should be reconsidered to include the benefits of preventing PIAT.

## Competing interests

The authors declare that they have no competing interests.

## Authors' contributions

Conception and designing of the study was done by JGP, PS, GD, and SEW. EV, GM, TM, GD, MF and SEW collected the data, while JGP, PS, TM and SEW analyzed the data. All authors wrote the manuscript. All authors read and approved the final manuscript.

## Pre-publication history

The pre-publication history for this paper can be accessed here:

http://www.biomedcentral.com/1471-2458/12/119/prepub
